# Effectiveness of one dose of killed oral cholera vaccine in an endemic community in the Democratic Republic of the Congo: a matched case-control study

**DOI:** 10.1016/S1473-3099(23)00742-9

**Published:** 2024-05

**Authors:** Espoir Bwenge Malembaka, Patrick Musole Bugeme, Chloe Hutchins, Hanmeng Xu, Juan Dent Hulse, Maya N Demby, Karin Gallandat, Jaime Mufitini Saidi, Baron Bashige Rumedeka, Moïse Itongwa, Esperance Tshiwedi-Tsilabia, Faida Kitoga, Tavia Bodisa-Matamu, Hugo Kavunga-Membo, Justin Bengehya, Jean-Claude Kulondwa, Amanda K Debes, Nagède Taty, Elizabeth C Lee, Octavie Lunguya, Justin Lessler, Daniel T Leung, Oliver Cumming, Placide Welo Okitayemba, Daniel Mukadi-Bamuleka, Jackie Knee, Andrew S Azman

**Affiliations:** a Department of Epidemiology, Johns Hopkins University, Baltimore, MD, USA; b Department of International Health, Johns Hopkins University, Baltimore, MD, USA; c Centre for Tropical Diseases and Global Health (CTDGH), Université Catholique de Bukavu, Bukavu, Democratic Republic of the Congo; d Department of Disease Control, London School of Hygiene & Tropical Medicine, London, UK; e Ministère de la Santé Publique, Hygiène et Prévention, Zone de Santé d'Uvira, Uvira, Democratic Republic of the Congo; f Oxfam DRC, Uvira, Democratic Republic of the Congo; g Rodolphe Merieux INRB-Goma Laboratory, Goma, North Kivu, Democratic Republic of the Congo; h Institut National de Recherche Biomédicale, INRB, Kinshasa, Democratic Republic of the Congo; i Ministère de la Santé Publique, Hygiène et Prévention, Division Provinciale de la Sante' Publique du Sud-Kivu, Bukavu, Democratic Republic of the Congo; j PNECHOL-MD, Community IMCI, Ministry of Health, Kinshasa, Democratic Republic of the Congo; k Service of Microbiology, Department of Medical Biology, University of Kinshasa, Kinshasa, Democratic Republic of the Congo; l University of North Carolina Population Center, University of North Carolina at Chapel Hill, Chapel Hill, NC, USA; m Department of Epidemiology, Gillings School of Global Public Health, University of North Carolina at Chapel Hill, Chapel Hill, NC, USA; n Division of Infectious Diseases and Division of Microbiology and Immunology, University of Utah School of Medicine, Salt Lake City, Utah, USA; o Geneva Centre for Emerging Viral Diseases and Division of Tropical and Humanitarian Medicine, Geneva University Hospitals, Geneva, Switzerland

## Abstract

**Background:**

A global shortage of cholera vaccines has increased the use of single-dose regimens, rather than the standard two-dose regimen. There is sparse evidence on single-dose protection, particularly in children. In 2020, a mass vaccination campaign was conducted in Uvira, an endemic urban setting in eastern Democratic Republic of the Congo, resulting in largely single-dose coverage. We examined the effectiveness of a single-dose of the oral cholera vaccine Euvichol-Plus in this high-burden setting.

**Methods:**

In this matched case-control study, we recruited individuals with medically attended confirmed cholera in the two cholera treatment facilities in the city of Uvira. The control group consisted of age-matched, sex-matched, and neighbourhood-matched community individuals. We recruited across two distinct periods: Oct 14, 2021, to March 10, 2022 (12–17 months after vaccination), and Nov 21, 2022, to Oct 18, 2023 (24–36 months after vaccination). Study staff administered structured questionnaires to all participants to capture demographics, household conditions, potential confounding variables, and vaccination status. The odds of vaccination for the case and control groups were contrasted in conditional logistic regression models to estimate unadjusted and adjusted vaccine effectiveness.

**Findings:**

We enrolled 658 individuals with confirmed cholera and 2274 matched individuals for the control group. 99 (15·1%) individuals in the case group were younger than 5 years at the time of vaccination. The adjusted single-dose vaccine effectiveness was 52·7% (95% CI 31·4 to 67·4) 12–17 months after vaccination and 44·7% (24·8 to 59·4) 24–36 months after vaccination. Although protection in the first 12–17 months after vaccination was similar for children aged 1–4 years and older individuals, the estimate of protection in children aged 1–4 years appeared to wane during the third year after vaccination (adjusted vaccine effectiveness 32·9%, 95% CI –30·7 to 65·5), with CIs spanning the null.

**Interpretation:**

A single dose of Euvichol-Plus provided substantial protection against medically attended cholera for at least 36 months after vaccination in this cholera-endemic setting. Although the evidence provides support for similar levels of protection in young children and others in the short term, protection among children younger than 5 years might wane significantly during the third year after vaccination.

**Funding:**

Wellcome Trust and Gavi, the Vaccine Alliance.

## Introduction

Safe water, sanitation, and hygiene (WASH) is the foundation of cholera prevention and control. Although universal access to safely managed WASH services remains the ultimate priority, this is probably a distant prospect.^1^ The killed whole-cell oral cholera vaccine (kOCV) is an effective short-term intervention to reduce cholera risk in high-burden settings and is a key component of the global roadmap to end cholera.^2^ kOCVs are typically delivered as a two-dose regimen that provides protection for at least 3 years.^3^, ^4^ In a meta-analysis of kOCV protection, the estimated two-dose efficacy was 58% (95% CI 42–69) over an average of 28 months after vaccination. Lower protection was noted among young children.^3^

The Euvichol-Plus vaccine (Eubiologics, Seoul, South Korea) is currently the only WHO-prequalified kOCV manufactured and included in the global stockpile after the Shanchol vaccine (Shantha Biotechnics, Hyderabad, India) ceased production in 2023.^5^ Euvichol-Plus is considered a bioequivalent of Shanchol.^6^ Almost all evidence of kOCV clinical protection is based on the studies carried out on Shanchol,^7^, ^8^, ^9^ although one observational study explored the protection conferred by a Euvichol-Plus two-dose regimen.^10^

Demand for kOCVs outstripped the global supply in 2022, with only 33 million of the 72 million requested doses distributed.^11^ In October, 2022, the International Coordinating Group, which manages the global emergency stockpile for cholera vaccines, suspended the provision of the standard two-dose regimen in emergency vaccination campaigns, replacing it with a single-dose regimen due to insufficient vaccine supply.^12^ However, there are few data on the protection offered by one dose of kOCV over extended periods (>12 months) or among children aged 1–4 years.

**Research in context**

**Evidence before this study**

In late 2022, due to increasing demand for killed, whole-cell, oral cholera vaccines (kOCVs) and insufficient production capacity, the International Coordinating Group (the organisation managing emergency stocks of kOCVs) changed policy to provide a single-dose regimen, rather than the standard two-dose regimen, for emergency vaccination campaigns. This decision was in line with WHO guidance on the use of a single dose in outbreaks, where short-term protection is key. However, this recommendation is based on few clinical studies, with short-term follow-up. There is also sparse evidence on the magnitude and duration of protection conferred by a single dose of kOCV, particularly in children younger than 5 years.

We searched PubMed for randomised trials and observational studies published in English before Nov 1, 2023, that reported estimates of protection conferred by a single dose of kOCV, using the term “(effectiveness OR efficacy) AND cholera* AND vaccine”. We found no published studies estimating the effectiveness of a single dose of Euvichol-Plus, and only one study reporting two-dose effectiveness. Despite this paucity of evidence, this is the only vaccine currently available in the global stockpile. To date, there has been one randomised trial conducted in Bangladesh between 2014 and 2016, and seven observational studies conducted between 2009 and 2016 in Guinea, Haiti, India, Malawi, Sudan, Zambia, and Tanzania (Zanzibar), reporting effectiveness estimates of a single dose of the current generation of kOCVs. Aside from the trial in Bangladesh, all estimates were based on secondary analyses that the studies were not powered to estimate. The Bangladesh trial is the only study to date that provides an age-stratified estimate of single-dose protection, and although it found an overall protective efficacy of 62% (95% CI 43 to 75) during the 2-year follow-up for individuals aged 5 years or older, it found no significant protection conferred by the Shanchol kOCV (a bioequivalent of Euvichol-Plus) for individuals younger than 5 years (protective efficacy –44%, 95% CI –220 to 35). Four of the seven observational studies provide single-dose vaccine effectiveness estimates only during the first 12 months after vaccination, with estimates ranging from 43% (95% CI –84 to 82) in Guinea to 93% (69 to 98) in Haiti. The three other observational studies providing a single-dose vaccine effectiveness estimate between 12 and 30 months after vaccination were unable to identify statistically significant protection conferred by kOCV, with estimates ranging between 32·5% (–318·0 to 89·1) in India and 40% (–31 to 73) in Haiti. No vaccine protection estimates have been published from the two identified cholera-endemic foci in Africa: the Democratic Republic of the Congo and Nigeria.

**Added value of this study**

In this vaccine effectiveness study, we show that a single dose of Euvichol-Plus vaccine can provide significant protection against medically attended cholera for at least 36 months after vaccination in a cholera-endemic setting in Africa, although the duration of protection in children younger than 5 years remains unclear. These estimates help fill crucial gaps in our understanding of the magnitude and duration of protection from a single dose of the most widely used kOCV, Euvichol-Plus and is one of only a few studies to measure protection in an endemic setting in Africa.

**Implications of all the available evidence**

The body of available evidence suggests that use of a single dose of kOCV in emergency situations where cholera is endemic, such as Uvira, provides similar protection against medically attended disease as two doses in the general population, at least over the first 2–3 years after vaccination. However, more evidence and analyses are needed to weigh the costs and benefits of tailored vaccination approaches for those younger than 5 years, including the possibility of providing a second dose at an earlier timepoint.

Only a few studies have estimated single-dose protection in the general population, with point estimates suggesting short-term protection up to 16 months after vaccination.^7^, ^8^, ^9^, ^13^, ^14^, ^15^, ^16^ A randomised trial in Bangladesh, the only study to provide age-stratified estimates of single-dose protection, suggested that Shanchol conferred no protection in children aged 1–4 years in the first 6 months after vaccination, despite significant protection for at least 2 years in individuals aged five years or older.^17^, ^18^

From July to September, 2020, the Ministry of Health of the Democratic Republic of the Congo conducted mass vaccination campaigns of Euvichol-Plus in the city of Uvira in the South Kivu province. The estimated coverage of the vaccination campaigns was low and, as most vaccinated individuals reported receiving only one dose, we assessed effectiveness of a single dose of kOCV during cholera outbreaks that occurred 12–17 months and 24–36 months after vaccination.

## Methods

### Study design

This matched case-control study was conducted in Uvira, a city of approximately 280 000 inhabitants on the northwestern shore of Lake Tanganyika with sporadic armed conflict, sociopolitical instability, and population displacement. Cholera infections are detected year round in Uvira, and have been since 1978, often with distinct seasonal peaks, and there have been notable historical outbreaks.^19^, ^20^ Household surveys conducted in 2016–17 in Uvira indicated surface water as the main drinking water source for 37·2% of households,^21^ and in areas close to the rivers and with the lowest tap water availability, more than 80% of households used drinking water contaminated with *Escherichia coli*.^22^ The same surveys estimated that 48·2% of the population relied exclusively on tap water for drinking needs,^21^ and a recent study showed that, between 2017 and 2021, the water service quality remained suboptimal or deteriorated in many parts of the city.^20^, ^23^

In April, 2020, severe flooding caused at least 54 deaths, the displacement of approximately 80 000 people, and substantial damage to housing and WASH infrastructure in Uvira, prompting the Ministry of Health to conduct emergency cholera vaccination campaigns.^24^ Vaccination took place in two rounds, from July 29 to Aug 8, 2020, and Sept 28 to Oct 5, 2020, targeting all individuals in Uvira aged 1 year and older. The campaigns included door-to-door vaccination for 5 days, followed by vaccination offered through health facilities. Although two rounds of vaccination were implemented, in a representative household survey we conducted 11 months after vaccination, 23% (95% CI 20–27) of the participants reported receiving two doses of the vaccine and 32% (95% CI 38–36) reported receiving one dose.^25^ No other kOCV has been administered in this population between the start of these campaigns and the end of our study period (Oct 18, 2023).

Ethical approvals were obtained from the Institutional Review Boards of the Johns Hopkins Bloomberg School of Public Health (reference number IRB00015785), the London School of Hygiene & Tropical Medicine (reference number 25365) and the École de Santé Publique at the University of Kinshasa (reference number ESP/CE/65/2021).

### Participants

This study is based on enhanced clinical surveillance of cholera implemented at the two official health facilities designated to treat patients with cholera in Uvira, the Cholera Treatment Centre at the Uvira General Referral Hospital, and the Cholera Treatment Unit at the Kalundu CEPAC health centre (to be referred to as CTCs).

Two cholera outbreaks occurred after mass vaccination, and we recruited individuals with laboratory-confirmed cholera during each outbreak, forming two distinct study periods. From Nov 21, 2022, to Jan 24, 2023, we retrospectively recruited matched control individuals for patients admitted to CTCs during the first outbreak (Oct 14, 2021, to March 10, 2022), approximately 12–17 months after the second round of mass vaccination campaigns (ie, study period 1). Between Oct 17, 2022, and Oct 18, 2023, individuals for the control group were recruited as they were admitted to the CTCs, 24–36 months after vaccination (ie, study period 2).

Study period 1 included all consenting individuals with suspected cholera who were aged at least 12 months during the vaccination campaigns, living in Uvira for the 2 weeks before admission to the CTC and during the 2020 vaccination campaigns, and who tested positive for cholera by culture or PCR. We aimed to recruit four individuals for the control group per individual with cholera, using high-resolution satellite imagery (appendix pp 2–3) to identify potential households for the control group on the same avenue (ie, the smallest administrative unit in Uvira) as the household for the case group. Households for the control group were then selected by simple random spatial sampling of digitised residential structures. Individuals were eligible for enrolment into the control group if: (1) they matched the age group (1–4 years, 5–9 years, 10–19 years, 20–39 years, 40–59 years or ≥60 years) and sex of the individual in the case group, (2) had not been admitted for acute watery diarrhoea or cholera in the 3 years before the case admission, (3) were living in Uvira in the 2 weeks before admission of the individual in the case group, (4) were living in Uvira at the time of the 2020 kOCV campaign and were eligible to be vaccinated, and (5) none of their household members reported being admitted to a formal health facility (as opposed to pharmacies, prayer homes, or traditional healers) for acute watery diarrhoea or cholera in the 4 weeks before admission of the individual in the case group (appendix pp 3–4). Age group was defined as the age on the first day of the second mass vaccination campaign round (Oct 1, 2020).

During study period 2, the case group inclusion criteria included the same age and residence criteria as in study period 1, but individuals had to test positive with both alkaline peptone water-enriched rapid diagnostic test and culture (performed at the onsite laboratory). We used enriched rapid diagnostic test results to help prioritise control recruitment due to insufficient human resources during the outbreak. In contrast to study period 1, for period 2 we conducted a home visit for each case within 3 days of hospital discharge to investigate the living and WASH conditions in each household and ascertain the vaccination status outside the hospital environment. We excluded individuals with cholera who died during a hospital stay and those whose residence could not be found during home visits. As in study period 1, four individuals were recruited for the control group from four randomly selected households in the same neighbourhood, although during this study period we selected households starting from the household of the case individual using the right-hand rule: from the front door of the household, study staff selected a random number between one and five, (ie, X), and walked to the Xth residential structure always turning right when faced with a barrier (appendix pp 2–3). In addition to the recruitment criteria used in study period 1, individuals in the control group were only eligible for enrolment in study period 2 if their household matched that of the individual in the case group in size (≤5 individuals, 6–10 individuals, and >10 individuals) and had at least one child younger than 5 years if the household for the case group had one. Age group had the same definition as for period 1.

Written informed consent was obtained from all participants aged 18 years and older, with written assent from individuals younger than 18 years in addition to written consent from their parent or guardian.

### Procedures

We attempted to identify and recruit all patients aged 12 months and older with acute watery diarrhoea within the 24 h before admission to the CTCs (referred to as suspected cholera). Trained health-care staff collected rectal swabs and stools from participants. Rectal swabs were enriched in alkaline peptone water for 6–18 h, depending on patient admission time. Specimens were tested for *Vibrio cholerae* by onsite rapid diagnostic tests and by culture, using standard methods (appendix p 1) at either an in-country reference laboratory (the Laboratoire Rodolphe Mérieux de l'Institut National de Recherche Biomédicale) in Goma (from Oct 17, 2021, to Sept 9, 2022), or at the onsite study laboratory (from Sept 10, 2022, onward). As PCR for the O1 serogroup of *V cholerae* was not available at either study laboratory, we shipped stool specimens (stool spotted on dry filter papers) for individuals with suspected cholera enrolled between Oct 14, 2021, and May 4, 2022, to Johns Hopkins University (Baltimore, MD, USA) for PCR detection of toxigenic *V cholerae* O1 following previously published methods.^26^

Study staff administered structured questionnaires to all participants (or their parent or guardian) to capture demographics, household conditions, potential confounding variables, and vaccination status. Before asking each individual whether they were vaccinated, study staff showed them photographs of the vaccine vials and of someone receiving the vaccine, in addition to explaining when and how the vaccines were delivered in Uvira, and how these might differ from other campaigns and routine vaccines. Participants reporting vaccination were asked the number of doses and when and where each one was taken. Vaccination cards were also used to verify vaccination status whenever possible. In study period 1, vaccine-related questions were asked to individuals in the case group in the clinic and in study period 2 they were asked both in the clinic and at a subsequent home visit. Any differences in the vaccination status reporting between the clinic and household interviews were solved through a third interview at the individual's house followed by a review of the data, discussion, and consensus within the study team. Potential confounders were identified based on a causal directed acyclic graph developed before the start of the study and we attempted to measure these through the interviews.

### Statistical analysis

The characteristics of participants in the case and control groups were compared using the standardised mean difference, which is the absolute difference in mean value between the individuals in the case and control groups divided by the pooled SD. In addition, we calculated p values from univariate conditional logistic regression models with case-control status as the dependent variable. In the primary analysis, we compared the odds of being vaccinated with a single kOCV dose between the case and control groups using conditional logistic regression models. Individuals reporting to have received two or more doses were excluded from the primary analyses. The vaccine effectiveness was calculated as 1 minus the estimated odds ratio (OR) for having received a single dose of vaccine, between the case and control groups. To produce consistent age group-specific and overall estimates of effectiveness across all ages, the conditional logistic regression model included an interaction term for age group (1–4 *vs* ≥5 years) and vaccination status. We derived the overall vaccine effectiveness estimate based on a linear combination of parameters from the two age groups, weighted by the proportion of cases within each, and estimated simultaneous CIs with the glht function in the multcomp R package.^27^ For continuous variables, we explored models using polynomials and restricted cubic splines, and compared them using Akaike Information Criterion. For combined estimates (ie, study period 1 and 2) and for those from study period 1 alone, we incorporated age as a continuous variable, separately by the two age groups (with a quadratic term for the age group ≥5 years), to adjust for potential confounding. For study period 2, estimates were adjusted for a set of potential confounders including a quadratic term for age, a restricted cubic spline for household size, household wealth index (as a continuous variable) derived from a principal component analysis of household assets ownership (appendix p 2), type of sanitation facility, whether the participant used a toilet shared by multiple households compared with using a private toilet, drinking water sources, and availability of a handwashing facility and soap. We also fit three alternative models with different sets of covariates to assess the robustness of the estimates (appendix pp 7–8).

In a secondary analysis, we estimated the vaccine effectiveness for at least one dose and two doses of kOCV compared with being unvaccinated. Point estimates and 95% CIs were primarily used to assess the weight of evidence and p values were considered statistically significant when they were less than 0·05. Analyses were performed in R (version 4.3.1).

### Role of the funding source

The funders of the study had no role in study design, data collection, data analysis, data interpretation, or writing of the report.

## Results

**Figure 1:**
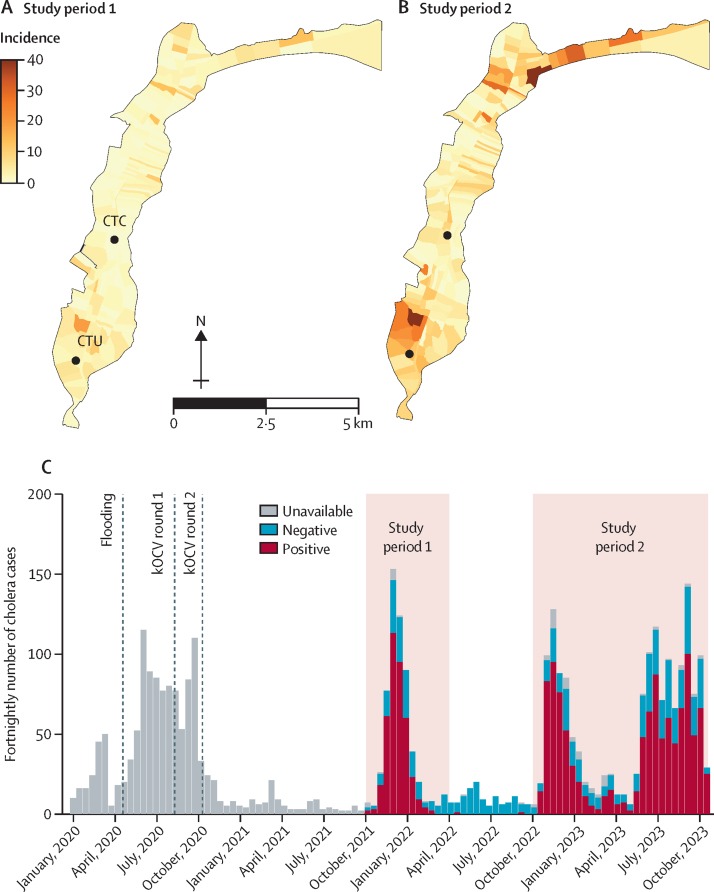
Number of patients with cholera admitted to cholera treatment facilities in Uvira (A) Represents study period 1. (B) Represents study period 2. (A) and (B) show the cumulative number of confirmed cases in each study period by neighbourhood (ie, avenue) across the city, with the locations of the two health facilities where patients were recruited. There were 14 patients with cholera living in neighbourhoods of Uvira not included in the official map borders who were not included in study period 2. The second outbreak (ie, study period 2) started in the northern part of the city and spread to a refugee camp where many residents were admitted to the CTC but not included in the study as they were not living in Uvira at the time of vaccination. (C) Shows the epidemic curve of patients with suspected and confirmed cholera admitted to the CTC at the Uvira General Referral Hospital and the CTU at the Kalundu CEPAC health centre. Cholera was confirmed by culture or PCR in study period 1, and by alkaline peptone water-enriched rapid diagnostic test and culture in study period 2. Among the 183 individuals with suspected cholera who were detected before study period 1, 146 (79·8%) were tested for *Vibrio cholerae* O1 by enriched rapid diagnostic test with 37 (25·3%) testing positive. CTC=cholera treatment centre. CTU=cholera treatment unit. KOCV=killed whole-cell oral cholera vaccine.

**Figure 2:**
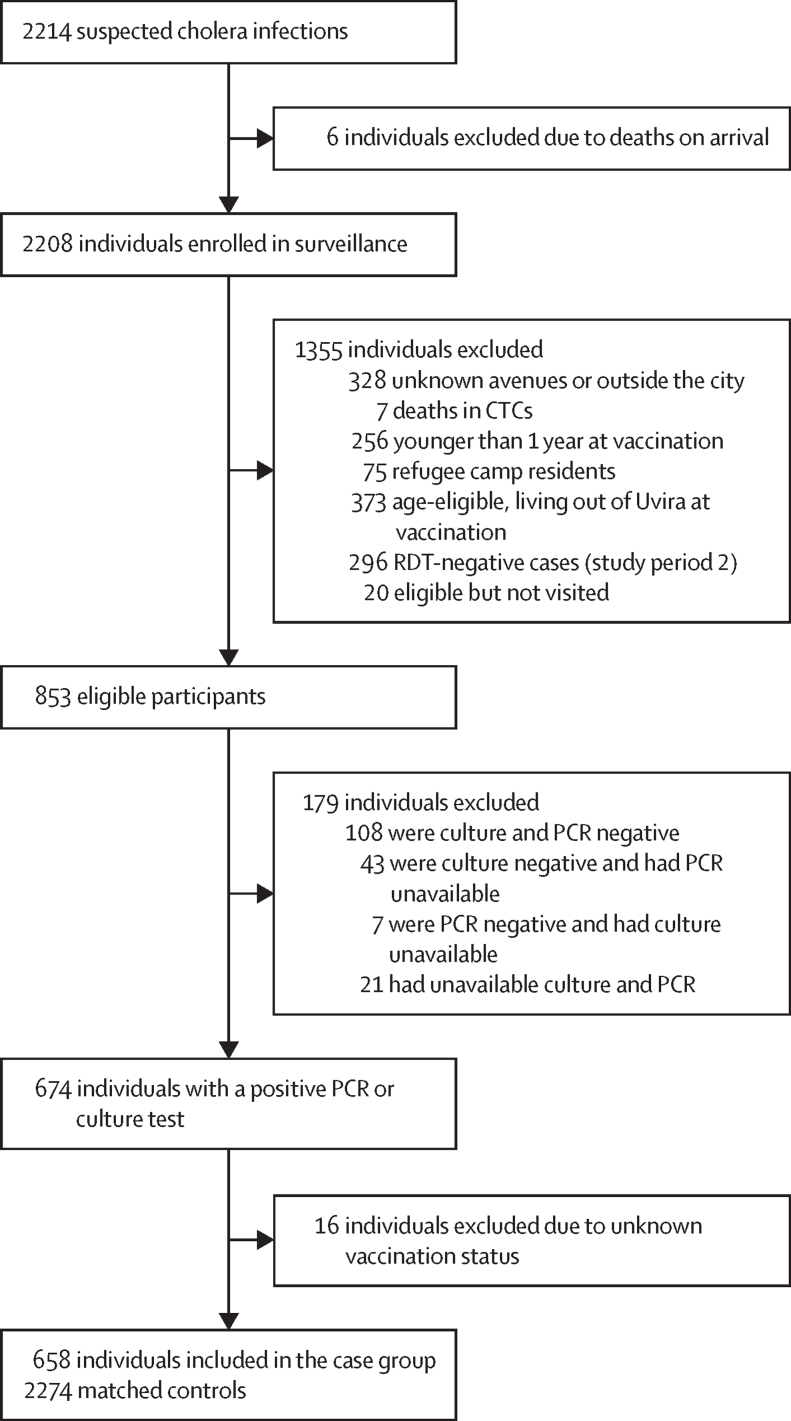
Flow chart of participant recruitment Individuals with unavailable culture results are those for whom suspected colonies were isolated, with positive oxidase test at the field laboratory, missing agglutination results due to an antiserum stockout, or for whom attempts to revitalise *Vibrio cholerae* O1 strains at the reference laboratory in Goma were unsuccessful. CTC=cholera treatment center. RDT=rapid diagnostic test.

Between Oct 14, 2021 to March 10, 2022, and Nov 21, 2022 to Oct 18, 2023, 658 unique individuals with confirmed cholera and 2274 matched individuals as controls were recruited (Figure 1, Figure 2), with 402 (61·1%) patients with cholera enrolled in the second period. The median age of participants at the time of the vaccination campaigns was 14·9 years (IQR 6·3–33·8) and 15·1% were younger than 5 years (table 1). 405 (61·6%) patients with cholera were recorded as severely dehydrated on admission. Patients with cholera were significantly older during the first study period compared with the second period (p=0·005), although they all had similar dehydration status (appendix p 5). Of the 537 culture-positive isolates, 390 (72·6%) were serotype Ogawa and the rest were Inaba. In study period 1, 109 (80·7%) of the 135 isolates were Ogawa and 26 (19·3%) were Inaba. In study period 2, 281 (69·9%) of the 402 isolates were Ogawa and 121 (30·1%) were Inaba.

**Table 1:** Characteristics of participants by case and control status

		**Case group**	**Control group**	**p value**	**Standardised mean difference**
Median age at vaccination (IQR)		14·1 (6·0–33·8)	15·1 (6·4–33·7)	0·0019	0·005
Age at vaccination		..	..	..	0·122
	1–4 years	99/658 (15·1%)	344/2274 (15·1%)	Ref	..
	5–9 years	146/658 (22·2%)	406/2274 (17·9%)	0·0080	..
	10–19 years	159/658 (24·2%)	605/2274 (26·6%)	0·72	..
	20–39 years	124/658 (18·8%)	482/2274 (21·2%)	0·84	..
	40–59 years	91/658 (13·8%)	310/2274 (13·6%)	0·42	..
	≥60 years	39/658 (5·9%)	127/2274 (5·6%)	0·45	..
Sex		..	..	..	0·001
	Female	337/658 (51·2%)	1166/2274 (51·3%)	Ref	..
	Male	321/658 (48·8%)	1108/2274 (48·7%)	0·86	..
Vaccination status		..	..	..	0·277
	Not vaccinated	452/658 (68·7%)	1280/2274 (56·3%)	Ref	..
	One dose	133/658 (20·2%)	718/2274 (31·6%)	<0·0001	..
	Two doses	73/658 (11·1%)	276/2274 (12·1%)	0·011	..
Vaccination card available (for those who were vaccinated)		29/206 (14·1%)	134/994 (13·5%)	0·11	0·017

Data are n/N (%) unless otherwise specified. p values are obtained from univariable conditional logistic regression models.

Overall, 133 (20·2%) patients with cholera reported receiving one dose of kOCV and 452 (68·7%) were unvaccinated. By comparison, 31·6% of matched controls reported receiving a single dose of kOCV and 56·3% were unvaccinated. Only 13·6% of vaccinated participants were able to show a vaccination card (table 1).

Additional data on sociodemographic and household characteristics were collected in study period 2 (table 2; appendix pp 6–7). Individuals in the case group were more likely to use toilets shared by multiple households than individuals in the control group (OR 1·41, 95% CI 1·11–1·79) and were less likely to live in houses with electricity (0·72, 0·56–0·92). Individuals in the case group were also more likely to live in households with lower wealth index, indicating a higher level of poverty (0·58, 0·44–0·76) than those in the control group. Although probably an artifact of hygiene kit distribution by the Uvira Health Zone that focused on case group households, we found that individuals in the case group were more likely to live in households with soap and water available for handwashing than those in the control group (1·86, 1·40–2·49).

**Table 2:** Characteristics of study participants in the vaccine effectiveness analysis 24–36 months after vaccination (study period 2)

		**Case group**	**Control group**	**p value**	**Standardised mean difference**
Age group at vaccination		..	..	..	0·179
	1–4 years	60/402 (14·9%)	235/1450 (16·2%)	Ref	..
	5–9 years	109/402 (27·1%)	292/1450 (20·1%)	0·056	..
	10–19 years	90/402 (22·4%)	376/1450 (25·9%)	0·30	..
	20–39 years	67/402 (16·7%)	283/1450 (19·5%)	0·074	..
	40–59 years	55/402 (13·7%)	190/1450 (13·1%)	0·43	..
	≥60 years	21/402 (5·2%)	74/1450 (5·1%)	0·44	..
Sex		..	..	..	0·005
	Female	215/402 (53·5%)	772/1450 (53·2%)	Ref	..
	Male	187/402 (46·5%)	678/1450 (46·8%)	0·95	..
Educational attainment*		..	..	..	0·353
	None or primary	52/126 (41·3%)	138/542 (25·5%)	Ref	..
	Lower secondary	21/126 (16·7%)	98/542 (18·1%)	0·028	..
	Upper secondary	46/126 (36·5%)	273/542 (50·4%)	0·0008	..
	Bachelor or higher	7/126 (5·6%)	33/542 (6·1%)	0·35	..
Occupation		..	..	..	0·113
	No work	71/402 (17·7%)	218/1450 (15·0%)	Ref	..
	Children younger than school age	45/402 (11·2%)	152/1450 (10·5%)	0·58	..
	Students	180/402 (44·8%)	642/1450 (44·3%)	0·71	..
	Informal work	92/402 (22·9%)	363/1450 (25·0%)	0·15	..
	Salaried	14/402 (3·5%)	74/1450 (5·1%)	0·043	..
	Missing	0/402	1/1450 (<0·1%)	..	..
Household size		7·0 (5·0 to 9·0)	7·0 (6·0 to 9·0)	0·3607	0·075
Drinking water source		..	..	..	0·033
	Improved	220/402 (54·7%)	817/1450 (56·3%)	Ref	..
	Unimproved	182/402 (45·3%)	632/1450 (43·6%)	0·46	..
	Missing	0/402	1/1450 (<0·1%)	..	..
Living in household with shared toilet		..	..	..	0·154
	Private	159/402 (39·6%)	684/1450 (47·2%)	Ref	..
	Shared	243/402 (60·5%)	766/1450 (52·8%)	0·0049	..
Toilet type		..	..	..	0·085
	Improved	262/402 (65·2%)	1003/1450 (69·2%)	Ref	..
	Unimproved	140/402 (34·8%)	447/1450 (30·8%)	0·11	..
Soap and water available for handwashing		217/402 (54·0%)	664/1450 (45·8%)	<0·0001	0·164
Living in household with electricity		150/402 (37·3%)	632/1450 (43·6%)	0·0090	0·128
Wealth index†		−5·3 (−50·0 to 31·3)	2·7 (−30·9 to 36·5)	0·0001	0·182
Vaccination status		..	..	..	0·244
	Not vaccinated	275/402 (68·4%)	850/1450 (58·6%)	Ref	..
	One dose	81/402 (20·2%)	444/1450 (30·6%)	<0·0001	..
	Two doses	46/402 (11·4%)	156/1450 (10·8%)	0·39	..
Vaccination card available (for those who were vaccinated)		22/123 (17·9%)	80/599 (13·4%)	0·025	0·125

Data are n/N (%) or median (IQR) unless otherwise specified. The distribution of age and sex for the case group and control group does not match perfectly because the number of individuals in the control group included for each individual in the case group varied slightly due to enrolled participants in the control group either not meeting the eligibility criteria (eg, not living in Uvira during vaccination) or not recalling their vaccination status. p values were obtained from univariable conditional logistic regression models.

* The question about education attainment was only asked to individuals aged ≥18 years.

† Wealth index was multiplied by 100. The higher the wealth index the richer the household.

Combining data from both study periods, 12–36 months after vaccination, we estimated an unadjusted single-dose vaccine effectiveness of 47·8% (95% CI 34·6–58·4) and after adjustment for age, the vaccine effectiveness was 48·2% (34·8–58·8).

In study period 1, 12–17 months after vaccination, the estimated unadjusted single-dose vaccine effectiveness was 54·4% (95% CI 34·4 to 68·3) and adjusted effectiveness was 52·7% (31·4 to 67·4). In study period 2, 24–36 months after vaccination, the estimated unadjusted vaccine effectiveness was 43·2% (24·0 to 57·6) and the adjusted vaccine effectiveness was 44·7% (24·8 to 59·4). The adjusted vaccine effectiveness for children aged 1–4 years was 73·5% (28·9 to 90·1) in study period 1, sharply declining to 32·9% (–30·7 to 65·5) in study period 2, and the confidence intervals spanned the null (table 3).

**Table 3:** Effectiveness of a single dose of oral cholera vaccine by age and time since vaccination

	**Case group**	**Control group**	**Unadjusted vaccine effectiveness (95% CI)**	**Adjusted vaccine effectiveness (95% CI)**
**12–36 months after vaccination**				
Overall	573 (419)	1998 (763)	47·8% (34·6 to 58·4)	48·2% (34·8 to 58·8)
1–4 years old	96 (61)	263 (129)	52·4% (22·5 to 70·8)	49·8% (15·6 to 70·2)
≥5 years old	463 (332)	1534 (584)	46·7% (31·8 to 58·4)	47·8% (32·8 to 59·5)
**12–17 months after vaccination (study period 1)**				
Overall	219 (170)	704 (300)	54·4% (34·4 to 68·3)	52·7% (31·4 to 67·4)
1–4 years old	34 (23)	83 (44)	68·3% (19·6 to 87·5)	73·5% (28·9 to 90·1)
≥5 years old	179 (141)	568 (243)	50·9% (28·0 to 66·6)	46·9% (21·0 to 64·3)
**24–36 months after vaccination (study period 2)**				
Overall	354 (249)	1294 (463)	43·2% (24·0 to 57·6)	44·7% (24·8 to 59·4)
1–4 years old	62 (38)	180 (85)	42·8% (−2·2 to 68·0)	32·9% (−30·7 to 65·5)
≥5 years old	284 (191)	966 (341)	43·4% (21·7 to 59·0)	47·5% (26·1 to 62·6)

Data are n (effective n) unless otherwise specified. Effective n is the sample size that effectively contributes to estimates of effectiveness in the conditional logistic regression models. Those participants in matched case-control sets where all people have the same vaccination status do not contribute to the estimates, nor do those who either do not know their vaccination status or the number of doses they received. For adjusted vaccine effectiveness, in study period 1 and in analyses combining data from both study periods, we only adjusted for age as a continuous variable.

In secondary analyses, the adjusted cumulative vaccine effectiveness across both study periods for at least one dose of kOCV was estimated to be 45·6% (95% CI 33·5–55·5; appendix pp 9–10). We were unable to reliably estimate two-dose vaccine effectiveness due to a low effective sample size, with post-hoc power calculations suggesting there was 50·9% power to detect significant vaccine protection when combining data from both study periods and 5·1% power in children younger than 5 years (appendix pp 10–11).

## Discussion

We found that a single dose of Euvichol-Plus kOCV provided protection against cholera for at least 36 months after vaccination. This study provides unique, policy-relevant insights into kOCV protection, assessing the single-dose effectiveness from the only available and most widely used cholera vaccine today, including estimates of the effectiveness for children aged 1–4 years and at discrete time periods after vaccination. Our results suggest that, at least in cholera-endemic areas such as Uvira, the use of one kOCV dose might provide significant protection for years rather than just months.

To date, one randomised trial^17^, ^18^ and seven observational studies have included estimates of one-dose protection of kOCVs.^7^, ^8^, ^9^, ^13^, ^14^, ^15^, ^28^ Six of these studies estimated short-term protection, which was measured for just a few months after vaccination and showed similar protection to two doses on this timescale. Two studies measured protection over a longer period, including a randomised trial in Bangladesh and a case-control study in Haiti, both using Shanchol. The Bangladesh trial was done over 2 years and estimated 54% (95% CI 16–75) efficacy in the first year and 67% (43–81) in the second year after vaccination among those aged 5 years and older.^17^ Secondary analyses from a case-control study in Haiti predicted that a single dose of Shanchol would confer 58% (4–82) protection at 16 months after vaccination, with the CIs including zero protection from 17 months after vaccination and onward.^14^ Our results are consistent with these previous studies in showing significant protection for the overall population for longer than a year, although Uvira, like Haiti (at the time of the study) and Bangladesh, is endemic for cholera so the first dose might have acted as a booster for previously infected individuals. More work is needed to characterise the epidemiological settings in which one dose might provide similar protection to that of the full regimen, and might include leveraging historic incidence rates of cholera or population-level immunological measures of previous exposures.^29^

Before this study, only one estimate of single-dose protection of a kOCV in children aged 1–4 years had been published.^17^, ^18^ This trial, in Bangladesh, suggested that young children did not benefit from a single dose of Shanchol, even during the first 6 months after vaccination.^17^, ^18^ By contrast, we found evidence that the population younger than 5 years in Uvira benefited from similar levels of protection to those aged 5 years and older at 12–17 months after vaccination; however, the point estimates dropped substantially in the third year after vaccination with CIs spanning the null. This observation is in line with studies showing similar levels of protection after natural infection between the two age groups.^30^ Discordant estimates between young children and older individuals have also been observed in kOCV studies with the full dose regimen,^3^ although there are only a handful of studies that present age-stratified estimates. Most studies have found lower effectiveness in young children but the difference in protection has been highly variable with large uncertainty (eg, ranging from no apparent difference in Viet Nam 10 months after vaccination,^31^ to 51% lower protective efficacy among children in Bangladesh 48 months after vaccination).^4^ The contrast of our estimates with other studies could be explained by several factors, including pre-existing population immunity and season of vaccination, as has been shown with other diseases,^32^ and differences both in gut microbiota composition and in prevalence of enteropathy. However, as in all observational studies, we cannot rule out confounding and selection bias.^33^, ^34^ Additional observational, and ideally randomised trials, would help further quantify the protection of one dose of kOCV among young children.

Although the focus of the study was to understand the direct effectiveness of kOCV, our analysis of potential risk factors highlighted key differences between individuals in the case group and matched individuals in the control group. Our results confirm the associations between cholera and markers of poverty, such as using a shared latrine, using an unimproved source for drinking water, and not having electricity in the household. Although kOCV provides protection against cholera, tackling fundamental risk factors such as access to safe water and sanitation are needed to sustainably control the disease.

This study comes with several limitations. First, like many previous kOCV effectiveness studies, the vaccination status was self-reported,^7^, ^14^, ^15^ and only 29 individuals in the case group (of the 206 reporting to be vaccinated) were able to provide a vaccination card. Measurement of the number of doses received months to years after a mass vaccination campaign is prone to recall bias, particularly in a place such as Uvira where mass vaccination campaigns targeting different pathogens are common. To minimise biases in classification of vaccination status, we used visual aids and a series of structured questions, and hospital and study staff reassured patients that their responses to the study questions would in no way affect their care. Although enumerators were not masked to the vaccination status of the individuals in the case group while enrolling the matched individuals into the control group, the vaccine coverage among the control group was similar to that measured in community coverage surveys (appendix p 12). Furthermore, sensitivity analyses restricted to only those with a vaccination card revealed similar effectiveness estimates (appendix p 7). There were slight differences in the protocol of the case-control study in each study period, which challenges the interpretability of the joint estimates from both periods. However, in sensitivity analyses simulating similar diagnostic criteria for individuals in the case group in study period 1 and study period 2, our qualitative findings remained consistent (appendix pp 11–12). The retrospective recruitment of the control group for the individuals admitted to the case group during study period 1 precluded adjustment for several individual and household factors that might have influenced cholera disease risk or vaccine acceptability. However, such factors are unlikely to have significantly influenced the magnitude of our vaccine effectiveness estimates, given the similarity of our overall estimates with those from study period 2 and previously published effectiveness studies.^7^, ^14^, ^15^ The retrospective nature of recruitment for the control group in study period 1 could also have led to differential recall of vaccination status among the case and control groups. Even in study period 2, our vaccine effectiveness estimates could still be confounded by unmeasured factors. Although the number of individuals younger than 5 years in the case group was higher than in most published vaccine effectiveness studies, our sample size in this important age group was still small and led to wide CIs around vaccine effectiveness estimates (appendix pp 5–6, 10–11). Finally, we were unable to obtain reliable estimates of two-dose protection, partly because few individuals in the case group reported receipt of two doses of kOCV due to low vaccination coverage in the population, and potentially also due to uncertainty in the reporting of more than one dose of kOCV (appendix pp 5–6, 10–11).

Data from dose-interval studies conducted in Cameroon and Zambia,^35^, ^36^ suggest that providing a second dose of kOCV a year or more after the first dose could lead to better and longer lasting protection against cholera than the current two-dose regimen, at least among individuals older than 5 years. Although more data are needed across different settings and for longer periods of time, our study extends the current evidence base on the protection of a single dose of kOCV, and, more specifically, on the protection of Euvichol-Plus, the most widely used cholera vaccine available today.

## Data sharing

Code and data from this study are available at https://github.com/HopkinsIDD/uvira_onedose_ocv_ve.

## Declaration of interests

We declare no competing interests.
