# Correction to *Lancet Infect Dis* 2024; published online Jan 18. https://doi.org/10.1016/S1473-3099(23)00742-9

**DOI:** 10.1016/S1473-3099(24)00068-9

**Published:** 2024-03

*Malembaka EB, Bugeme PM, Hutchins C, et al. Effectiveness of one dose of killed oral cholera vaccine in an endemic community in the Democratic Republic of the Congo: a matched case-control study.* Lancet Infect Dis *2024; published online Jan 18. https://doi.org/10.1016/S1473-3099(23)00742-9*—The appendix of this Article has been corrected as of Jan 29, 2024.

